# Advanced Glycation End Products Upregulate Insulin Receptor Substrate-1 (IRS-1) in Human Cumulus Granulosa Cells

**DOI:** 10.3390/cells15131174

**Published:** 2026-06-28

**Authors:** Zaher Merhi

**Affiliations:** 1 Department of Obstetrics and Gynecology, Division of Reproductive Endocrinology and Infertility, Albert Einstein College of Medicine, Bronx, NY 10461, USA; zaher.merhi@rfcfertility.com; 2 Department of Obstetrics and Gynecology, Division of Reproductive Endocrinology and Infertility, Maimonides Medical Center, Brooklyn, NY 11219, USA; 3 Division of Reproductive Endocrinology and Infertility, Rejuvenating Fertility Center, New York, NY 10019, USA

**Keywords:** advanced glycation end-products, granulosa cells, insulin, IRS-1

## Abstract

Women with polycystic ovary syndrome (PCOS) and insulin resistance (IR) commonly have elevated serum advanced glycation end-products (AGEs) that accumulate in their ovaries, potentially altering ovarian function. AGEs have been shown to interfere with insulin signaling in granulosa cells (GCs) by suppressing the translocation of glucose transporters. Additionally, PCOS has been associated with insulin receptor substrate (IRS)-1 polymorphisms and upregulation in IRS-1 gene expression in GCs. We hypothesize that AGEs are partly responsible for ovarian IR by altering IRS-1 in human GCs. Cumulus granulosa cells from women undergoing IVF were cultured in control media or media containing human glycated albumin (HGA) as a source of AGEs. The quantification of mRNA expression was compared using RT-PCR for receptors for AGEs (RAGE), IRS-1, IRS-2, Glucose Transporter Type (GLUT)-1, and nuclear factor kappa-light-chain-enhancer of activated B cells (NF-κB). In addition, IRS-1 protein intensity was assessed by immunofluorescence. Compared to the control group, HGA-treated GCs showed a statistically significant upregulation in RAGE mRNA by 314% and in IRS-1 mRNA by 423%. Even though there was a 183% increase in GLUT-1 mRNA, it did not reach statistical significance. There was no change in IRS-2 or NF-κB mRNA expression levels. Immunofluorescence showed that IRS-1 deposition was visualized in GCs, and the addition of HGA resulted in a significant increase in the intensity of IRS-1 protein compared to control cells. The AGE-induced upregulation in IRS-1, the primary insulin receptor substrate, in GCs could indicate compensation for insulin action deficiency and potentially IR. Additionally, immunofluorescence analysis of GLUT-4 revealed a statistically significant reduction in cytoplasmic GLUT-4 signal in HGA-treated GCs compared to controls (2.10 ± 0.21 vs. 3.81 ± 0.22 arbitrary units; *p* = 0.010), consistent with AGE-mediated disruption of glucose transporter localization. These results suggest that AGE exposure may initiate early molecular changes consistent with altered insulin signaling, but do not establish insulin resistance per se.

## 1. Introduction

Modern dietary patterns, particularly the consumption of precooked fast-food meals and foods rich in protein and fat such as meat, cheese, and egg yolk, contain high levels of advanced glycation end-products (AGEs), leading to elevated serum concentrations of AGEs following their ingestion [1,2]. Advanced glycation end-products are a diverse group of highly reactive molecules known for their proinflammatory potential [3]. Elevated levels of AGEs in the bloodstream and the ovarian tissue have been associated with polycystic ovary syndrome (PCOS) [4].

At the molecular level, advanced glycation leads to irreversible cross-linking of proteins with consequent loss of structural integrity and biological function [5,6,7]. AGEs exert cellular damage through multiple mechanisms [8,9]. First, they promote cross-link formation between key structural molecules within the extracellular matrix basement membrane, most notably collagen, thereby compromising tissue architecture [1]. Second, AGE binding to its receptor RAGE activates downstream inflammatory signaling cascades and promotes apoptosis [2]. In contrast, the soluble decoy receptor for AGEs (sRAGE) exerts opposing, anti-inflammatory effects [5]. sRAGE is a truncated, extracellularly secreted isoform of RAGE [10] that is detectable in both peripheral blood and ovarian follicular fluid [11,12,13,14]. By sequestering circulating AGEs, sRAGE competitively inhibits the engagement of the proinflammatory AGE-RAGE signaling axis, thereby attenuating its downstream intracellular consequences [3,10]. Unlike membrane-bound RAGE, sRAGE is widely regarded as a protective, “counter-regulatory” receptor [10,15,16,17], and circulating sRAGE levels have been investigated as a predictive biomarker for the development and progression of a range of conditions, including diabetes mellitus, cardiovascular disease, endothelial dysfunction, and pulmonary disease [18,19,20,21]. Within the context of ovarian physiology, our group has previously demonstrated that follicular fluid sRAGE levels correlate positively with established markers of ovarian reserve [14].

Ovarian AGE levels are influenced by endogenous and exogenous sources, such as diet and smoking [1,22]. Feeding animals a high-AGE diet can induce insulin resistance and elevated serum testosterone (T) levels and ovarian weight [23], leading to metabolic and hormonal disturbances that imitate the phenotype observed in PCOS [24]. Corroborating these findings in animals, a clinical study demonstrated that adherence to a low-AGE diet in women with PCOS resulted in significant reduction in serum AGE levels, T levels, and markers of insulin resistance [2]. Despite accumulating evidence implicating AGE accumulation in the ovarian tissue of women with PCOS [4] and their established contribution to several PCOS-associated comorbidities [25], the mechanistic links underlying AGE-mediated ovarian dysfunction remain poorly defined.

Research exploring the impact of elevated circulating AGEs on the pathology of PCOS, a prevalent metabolic and reproductive disorder, has identified the presence of AGE-modified proteins and RAGE within human ovarian tissue [4]. Notably, a distinct qualitative pattern of AGE, RAGE, and the signaling mediator nuclear factor kappa-light-chain-enhancer of activated B cells (NF-κB) was detected in the granulosa cell (GC) layer of PCOS ovaries compared to controls, suggesting localized effects within the ovaries. AGEs have been shown to interfere with insulin signaling in GCs by suppressing the translocation of the Glucose Transporter Type 4 (GLUT-4) [26]. Given that insulin receptor substrate-1 (IRS-1) polymorphisms and upregulation of IRS-1 gene expression in GCs have been associated with PCOS [27,28], we thus hypothesize that AGEs are partly responsible for ovarian insulin resistance by altering IRS-1 expression.

## 2. Methods

### 2.1. Participants

Informed consent was obtained from all participants, and the study received approval from the Institutional Review Board (IRB# M13-062 and i6902). A total of 8 (*n* = 4 per experiment) infertile women, aged between 34 and 42 years, undergoing in vitro fertilization (IVF) for infertility were recruited. Eligibility criteria included having a normal ovarian reserve, characterized by day 3 follicle-stimulating hormone (FSH) levels below 10 mIU/mL and antral follicle count by transvaginal ultrasound of more than 10. Because the aim was to assess whether AGEs induce a PCOS-like phenotype, women diagnosed with PCOS according to the Rotterdam criteria were excluded from the study.

No participant had a history of diabetes, endometriosis, thyroid disease, or metabolic disorders. Standard metabolic screening (fasting glucose and lipid profile) was performed to exclude metabolic abnormalities. None of the participants had prediabetes or impaired glucose tolerance, confirmed by normal fasting glucose values. No participant was taking metformin or any other insulin-sensitizing agent at the time of the study. All patients underwent conventional ovarian stimulation using gonadotropins. When two or more ovarian follicles reached a diameter of 18 mm or greater, human chorionic gonadotropin (hCG) was administered to induce oocyte maturation, followed by transvaginal ultrasound-guided oocyte retrieval approximately 34–36 h later.

### 2.2. Cumulus Granulosa Cell Collection and Culture for Reverse Transcription-PCR (RT-PCR) Analysis

In this study, we focused on cumulus GCs that express characteristics distinct from mural GCs [29,30]. Cumulus cells communicate with each other and with the oocyte through specialized gap junctions that allow metabolic exchange and transport of signaling molecules for appropriate follicular development [31,32]. Thus, cumulus GCs arguably play a more important role in regulating oocyte maturation and in reflecting oocyte quality [31,32].

Cumulus GCs from 4 participants were collected on the day of the oocyte collection. Following the identification of the cumulus–oocyte complex within the aspirate, cumulus GCs were mechanically isolated by carefully cutting away the cumulus layer from each oocyte. The GCs obtained from each participant were pooled, suspended, then briefly treated with 0.614 U/mL of hyaluronidase (Sigma, St. Louis, MO, USA). The final pellet of GCs was washed again with fresh medium composed of DMEM-F12 and 1% fetal bovine serum (FBS). Because naturally occurring glycated albumin is a sensitive marker of glycemic control, commercially available human glycated albumin (HGA) was used throughout the study as a precursor for AGEs, consistent with previous research [26,33].

The GCs from each participant were divided into two equal groups and cultured in 24-well plates pretreated with poly-l-lysine for 5 min. Each participant’s GCs were treated under two different conditions: either the control group treated with media alone as control or the treatment group exposed to HGA (0.4 mg/mL, Sigma Aldrich, St. Louis, MO, USA) for 24 h. The dose of HGA was chosen based on previous research [26,33].

RNA extraction was performed using Trizol and chloroform. The quality of the isolated RNA was assessed using a Nanodrop Spectrophotometer and an Agilent Bioanalyzer (Santa Clara, CA, USA). Only samples with a minimum concentration of 10 ng/μL and an optical density (OD) 260/280 ratio ranging from 1.8 to 2.0 were included for analysis. All 4 samples collected for RT-PCR analysis met these quality criteria and were included in the final analysis. Gene expression levels of RAGE, IRS-1, IRS-2, GLUT-1, and NF-κB were quantified. Reverse transcription-PCR was carried out using SYBR Green I chemistry. The primers used were synthesized by Fisher (Pittsburgh, PA, USA). Glyceraldehyde-3-phosphate dehydrogenase (GAPDH) was employed as a loading control, and relative gene expression levels were calculated using the 2^−ΔΔCT^ method.

### 2.3. Immunofluorescence Analysis for IRS-1 in Human Cumulus Granulosa Cells

Cumulus GCs from an additional 4 participants were used for immunofluorescence analysis. For each participant, the GCs were divided into two equal groups and cultured on eight-well culture slides (Permanox^®^ slides, 0.8 cm^2^/well, sterile, 96/cs; Nunc^®^ Lab-Tek^®^ Chamber Slide™ system, Sigma, St. Louis, MO, USA). One group of cells was treated with culture media alone as a control, while the other group was treated with HGA (0.4 mg/mL) for 24 h. The GCs were then fixed using 3.7% formaldehyde and permeabilized with 0.2% Triton X-100 for 10 min at room temperature. To prevent non-specific binding, the cells were blocked with 1% bovine serum albumin (BSA) in PBS for 1 h. After blocking, the cells were incubated overnight at 4 °C with a primary antibody targeting IRS-1 (1:300 dilution; Cell Signaling Technology Inc., Danvers, MA, USA).

Following incubation, the cells were thoroughly washed and treated with a secondary antibody (1:200 dilution; Alexa Fluor 488 goat anti-rabbit IgG; Invitrogen, Waltham, MA, USA) for 1–2 h at room temperature. The nuclei were stained using DAPI mounting media (Vector Labs, Inc., Burlingame, CA, USA) and visualized under a Zeiss 510 META Laser Scanning Confocal Microscope. Images were captured with a Plane-NEOFLUAR 25× Immersion objective lens. For image analysis, MetaMorph software version 7.7 was used with a threshold of 62 (on a 0–255 intensity scale) to eliminate background signals generated by DAPI. All images were acquired using identical laser power, gain, and detector settings across control and HGA-treated conditions, and no differential post-acquisition brightness or contrast adjustments were applied between groups. The intensity of individual cells was measured by subtracting nuclear staining from cell surface staining.

### 2.4. Immunofluorescence Analysis for GLUT-4 in Human Cumulus Granulosa Cells

GLUT-1 and GLUT-4 serve distinct roles in glucose transport in granulosa cells: GLUT-1 mediates constitutive, insulin-independent basal glucose uptake and was therefore assessed at the mRNA level as a marker of basal transcriptional response to AGE exposure (Section 2.2), whereas GLUT-4 is the principal insulin-responsive transporter whose subcellular localization reflects downstream IRS-1/PI3K/Akt signaling competence and was therefore assessed at the protein and localization level. To assess the effect of AGEs on GLUT-4 protein expression and localization, immunofluorescence analysis was performed on cumulus GCs from the same four participants used for IRS-1 immunofluorescence. GCs were cultured as described in Section 2.3 under control conditions or with HGA (0.4 mg/mL) for 24 h. Following fixation and permeabilization, cells were incubated overnight at 4 °C with a primary antibody targeting GLUT-4 (Glucose Transporter Type 4; 1:300 dilution; Alexa Fluor 488 goat anti-rabbit IgG secondary antibody; Invitrogen). Nuclei were counterstained with DAPI mounting media. Images were acquired using the Zeiss 510 META Laser Scanning Confocal Microscope with a Plane-NEOFLUAR 25× Immersion objective lens. Quantitative image analysis was performed using MetaMorph software. Nuclear (DAPI) masks were generated by thresholding the blue channel, and a perinuclear cytoplasmic region of interest was defined by radial dilation of each nuclear mask. Background-corrected GLUT-4 fluorescence intensity (green channel) was measured separately within nuclear and cytoplasmic compartments for each cell, and mean intensities were compared between HGA-treated and control groups.

### 2.5. Statistical Analysis

Comparisons were made using paired *t*-tests. For cell culture experiments, a multilevel (hierarchical) analysis was employed to address the clustering of cells from the same participant but exposed to different conditions. This approach ensured that standard errors were adjusted to avoid inflating the type I error rate. The statistical model treated the “condition” as a fixed effect and the “participant” as a random effect. For RT-PCR and immunofluorescence experiments, a sample size of 4 was considered sufficient to detect statistical differences with 80% power and a two-tailed α error of 0.05. RT-PCR results were reported as relative copy numbers ± standard error of the mean (SEM), with controls standardized to a value of 1. Immunofluorescence results were determined by averaging the densitometry values for each treated cell group, then data were expressed as mean ± SEM. All statistical analyses were conducted using GraphPad Prism 10, with a significance threshold set at *p* ≤ 0.05.

## 3. Results

The demographics and clinical characteristics of the study participants are summarized in Table 1. Study participants had a mean age of 35.7 ± 1.2 years and a mean BMI of 25.6 ± 1.3 kg/m^2^, consistent with a normal-weight population. Baseline ovarian reserve parameters were within normal range, with a mean day 3 FSH of 7.3 ± 0.9 mIU/mL. Participants underwent controlled ovarian stimulation for a mean of 11.4 ± 0.6 days, requiring a mean total gonadotropin dose of 3258 ± 364 IU per cycle, and a mean of 12.8 ± 1.4 oocytes were retrieved per participant.

### 3.1. Effect of HGA on mRNA in Human Cumulus Granulosa Cells

**Figure 1:**
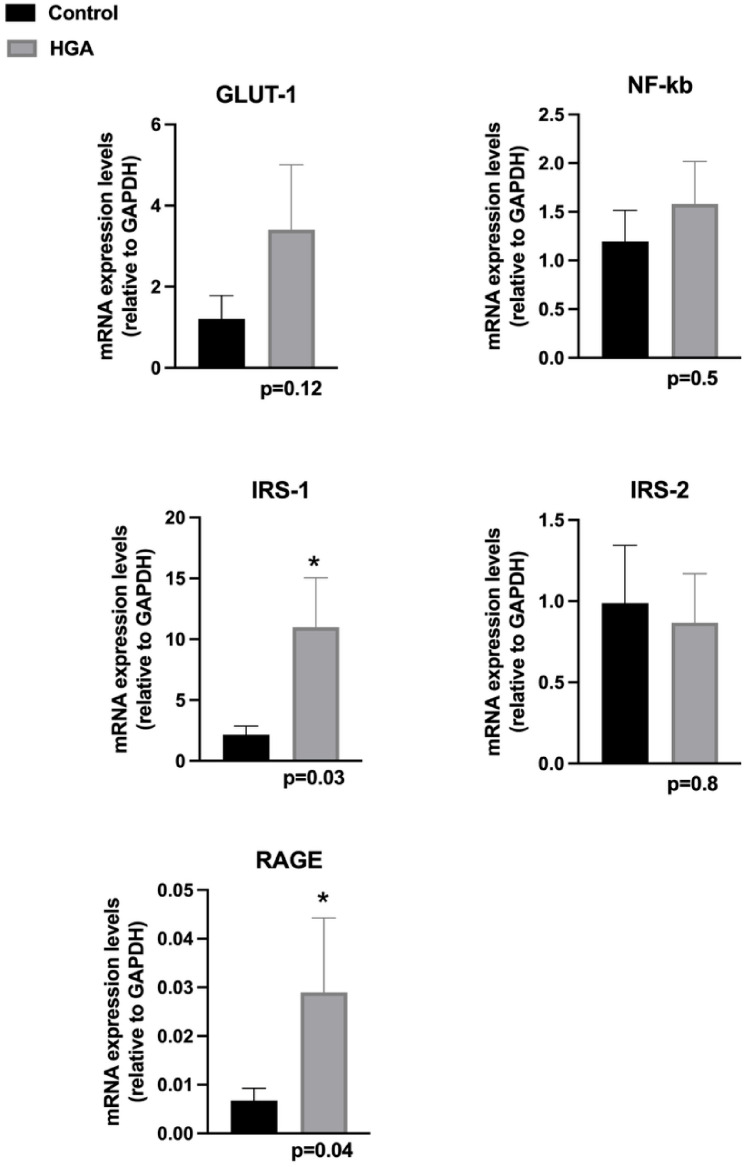
Effect of human glycated albumin (HGA), as a precursor for AGEs, on mRNA expression levels relative to GAPDH by RT-PCR for GLUT-1, NF-κB, IRS-1, IRS-2, and RAGE in human cumulus granulosa cells (GCs). Pooled cumulus GCs of women (*n* = 4) undergoing oocyte retrieval following ovarian stimulation for IVF were mechanically collected by cutting the cumulus layer from each oocyte. Cells were treated with HGA (0.4 mg/mL) or with media alone as control for 24 h after which cell RT-PCR was performed. Data are expressed as relative copy numbers ± standard error of the mean; controls were standardized to a value of 1.0. * *p* < 0.05 indicates a statistically significant difference between HGA treatment and media alone.

Exposure to HGA produced selective and statistically significant changes in gene expression compared to cells cultured in media alone (Figure 1). RAGE mRNA was upregulated by approximately 314% (*p* = 0.04), consistent with a transcriptional response to the AGE stimulus and indicative of enhanced AGE-receptor signaling capacity within the GCs. IRS-1 mRNA expression was upregulated by approximately 423% (*p* = 0.03), representing the most pronounced transcriptional change observed across all targets assessed. In contrast, IRS-2 mRNA expression showed no significant change (*p* = 0.8), suggesting that the AGE-mediated effect on insulin receptor substrate expression is member-specific among the IRS family, selectively targeting IRS-1. Similarly, NF-κB mRNA expression was not significantly changed (*p* = 0.5), indicating that the transcriptional response to HGA under these experimental conditions did not activate the classical NF-κB inflammatory pathway at the mRNA level. Although GLUT-1 mRNA expression showed a 183% increase in HGA-treated cells, this did not reach statistical significance (*p* = 0.12), possibly due to the limited sample size or reflecting a downstream effect requiring longer exposure or higher AGE concentrations.

### 3.2. Effect of HGA on IRS-1 Protein Intensity in Human Cumulus Granulosa Cells

**Figure 2:**
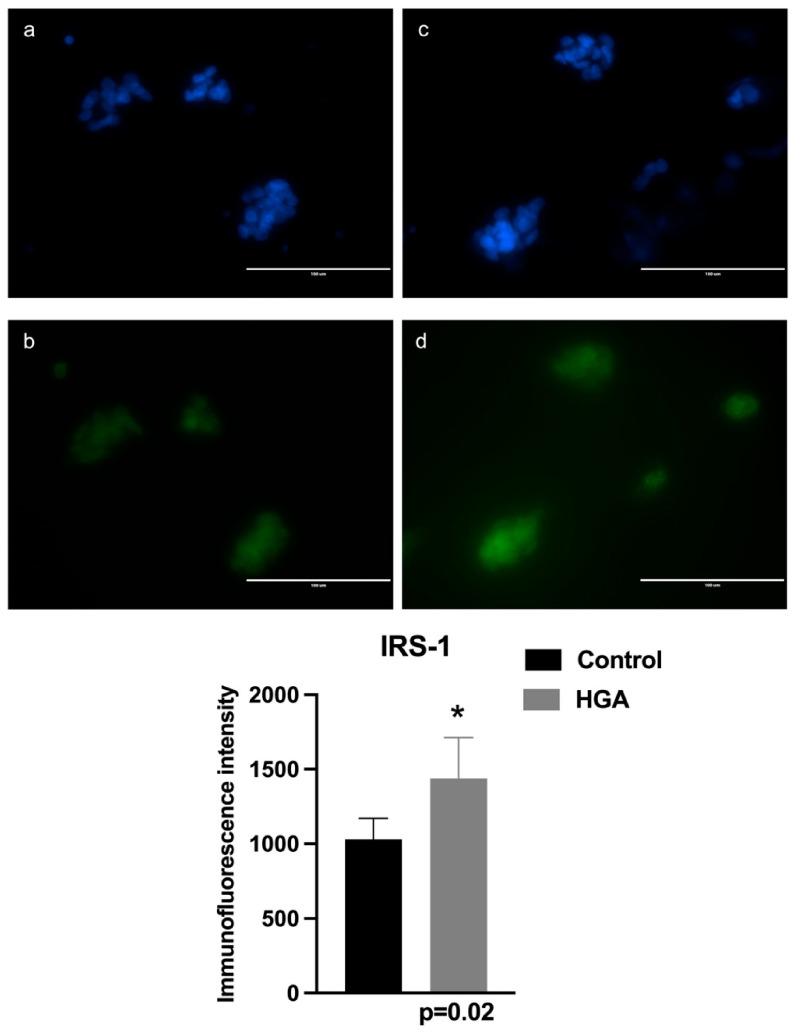
Effect of human glycated albumin (HGA), as a precursor for AGEs: IRS-1 protein intensity in human cumulus granulosa cells (GCs). Pooled cumulus GCs of women (*n* = 4) undergoing oocyte retrieval following ovarian stimulation for IVF were mechanically collected by cutting the cumulus layer from each oocyte. Immunofluorescence images display positive IRS-1 signals in green (**b**,**d**), while nuclei are stained blue with DAPI (**a**,**c**). Images a and b represent the control group while images c and d show cells treated with HGA. Data were determined by averaging the densitometry values for each treated cell group, then were expressed as mean ± SEM. * *p* < 0.05 indicates a statistically significant difference between HGA treatment and media alone.

To confirm the mRNA findings at the protein level, IRS-1 expression was assessed by immunofluorescence in an independent set of four participants (Figure 2). IRS-1 immunoreactivity was clearly detectable in GCs under both conditions, confirming constitutive expression of this protein in human cumulus GCs. HGA treatment resulted in a statistically significant increase in IRS-1 protein intensity compared to control cells (1440 ± 273 vs. 1029 ± 142 arbitrary units; *p* = 0.02), representing an approximately 40% increase in IRS-1 protein deposition. The concordance between elevated IRS-1 mRNA and increased IRS-1 protein intensity provides complementary evidence that AGE exposure upregulates IRS-1 at both the transcriptional and translational levels in human cumulus GCs.

### 3.3. Effect of HGA on GLUT-4 Protein Intensity in Human Cumulus Granulosa Cells

**Figure 3:**
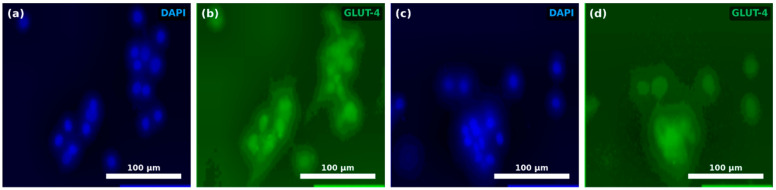
Representative immunofluorescence images of GLUT-4 (Alexa Fluor 488, green) and nuclei (DAPI, blue) in human cumulus granulosa cells under control and HGA-treated (0.4 mg/mL, 24 h) conditions. (**a**) Control DAPI; (**b**) control GLUT-4; (**c**) HGA DAPI; (**d**) HGA GLUT-4. All images were acquired at 40× magnification under identical laser power, gain, and detector settings, with no differential post-acquisition adjustments applied between groups.

To provide functional context for the observed IRS-1 upregulation, GLUT-4 protein expression and subcellular distribution were assessed by immunofluorescence in an independent set of four participants (Figure 3). GLUT-4 immunoreactivity was detectable in GCs under both conditions. Quantitative analysis of background-corrected fluorescence intensity revealed a statistically significant reduction in cytoplasmic GLUT-4 signal in HGA-treated cells compared to controls (2.10 ± 0.21 vs. 3.81 ± 0.22 arbitrary units; *p* = 0.010), representing a 45% decrease in perinuclear cytoplasmic GLUT-4 intensity. In contrast, nuclear GLUT-4 signal did not differ significantly between groups (6.07 ± 0.59 vs. 6.76 ± 0.29 arbitrary units; *p* = 0.385). Whole-cell GLUT-4 intensity did not reach statistical significance either (4.67 ± 0.43 vs. 5.20 ± 0.24 arbitrary units; *p* = 0.426). The selective reduction in cytoplasmic GLUT-4 signal in HGA-treated cells, in the absence of a significant change in whole-cell or nuclear signal, is consistent with impaired cytoplasmic GLUT-4 availability and supports AGE-mediated disruption of glucose transporter localization in human cumulus GCs.

## 4. Discussion

The present study provides novel evidence that AGEs significantly upregulate both mRNA and protein expression of IRS-1 in human cumulus GCs. IRS-1 serves as a key adaptor protein in insulin signaling, mediating downstream effects on glucose metabolism and cellular function [34]. These findings build upon previous research showing that women with PCOS exhibit elevated AGEs [35] and altered insulin signaling in GCs, particularly involving glucose transporter regulation and IRS-1 expression [26,27,28].

Our results may suggest that AGEs may contribute to ovarian insulin resistance by directly modulating IRS-1 levels. While the upregulation of IRS-1 could be interpreted as a compensatory mechanism to counteract diminished insulin signaling, chronic overexpression—especially in the context of dysregulated phosphorylation states—may exacerbate post-receptor signaling defects, as seen in muscle and adipose tissues of women with PCOS [36]. Specifically, aberrant serine phosphorylation of IRS-1 at residues such as Ser307 has been implicated in impaired insulin signaling, leading to dysregulated glucose uptake and disrupted steroidogenesis [37].

This proposed hypothesis may align with accumulating evidence that ovarian insulin resistance contributes to anovulation and hormonal imbalance in PCOS. Notably, our model employed GCs from non-PCOS women, suggesting that AGE-induced IRS-1 alterations may represent an early or independent pathway toward insulin resistance in ovarian tissue, even in the absence of underlying PCOS pathology. This may have implications for women with metabolic syndrome, diabetes, or high dietary AGE intake, where ovarian function could be compromised before overt reproductive symptoms develop.

Complementing the IRS-1 mRNA and protein findings, immunofluorescence analysis of GLUT-4 in HGA-treated GCs revealed a statistically significant reduction in cytoplasmic GLUT-4 signal (*p* = 0.010), consistent with impaired glucose transporter availability at the subcellular level. GLUT-4 is an insulin-responsive glucose transporter whose translocation from intracellular vesicles to the plasma membrane is a key downstream effector of IRS-1/PI3K/Akt signaling. In insulin-sensitive tissues, this translocation is impaired when IRS-1 undergoes aberrant serine phosphorylation, leading to reduced PI3K/Akt activation and failure of GLUT-4 vesicles to reach the cell surface. The present finding that cytoplasmic GLUT-4 signal is reduced in AGE-exposed GCs, in the context of concomitant IRS-1 upregulation, supports the hypothesis that AGEs may disrupt the downstream insulin signaling cascade in human cumulus GCs, resulting in impaired glucose transporter mobilization. These data provide preliminary functional evidence for the mechanistic model proposed in this study and are consistent with prior work by Diamanti-Kandarakis et al. (26), who demonstrated that AGEs suppress GLUT-4 translocation in granulosa cells. While the present immunofluorescence approach does not distinguish static GLUT-4 localization from insulin-stimulated dynamic translocation, the observed reduction in cytoplasmic signal may reflect baseline redistribution of GLUT-4 in response to AGE-induced signaling disruption, a finding that warrants further investigation under insulin-stimulated conditions. Western blot analysis could have provided a more quantitative measure of protein changes. Additionally, isotype IgG controls were not included to formally validate secondary antibody specificity at the dilution used, and the possibility that non-specific secondary antibody binding contributed to the nuclear immunoreactivity observed for IRS-1 and GLUT-4 cannot be formally excluded; future studies should incorporate matched isotype controls. The immunofluorescence images were acquired using a 25× immersion objective, which provides adequate optical sectioning but limits resolution of fine subcellular detail; higher-magnification objectives (e.g., 40× or 63× oil immersion) would better resolve cytoplasmic versus nuclear localization in future work. Finally, GLUT-4 mRNA was not measured in parallel with GLUT-4 protein localization; future studies should assess GLUT-4 at both the transcriptional and protein levels to provide a more complete picture of glucose transporter regulation. Finally, the HGA dose and exposure time were based on prior studies but may not fully reflect physiological conditions.

Several limitations merit consideration. First, we utilized luteinized cumulus GCs obtained after gonadotropin stimulation, which may not fully represent non-luteinized or mural GCs; these cells have distinct metabolic and signaling profiles. Second, sample size constraints limited our ability to perform dose–response experiments or to analyze follicles of different sizes separately—an important consideration since follicular maturity may influence GC sensitivity to AGEs. Third, while IRS-1 expression changes were quantified, functional downstream events such as phosphorylation status, PI3K/Akt or MAPK activation, and insulin-stimulated GLUT-4 translocation were not assessed under insulin-stimulated conditions. Notably, immunofluorescence analysis of GLUT-4 protein localization in the present study revealed a significant reduction in cytoplasmic GLUT-4 signal following HGA exposure (*p* = 0.010), providing preliminary evidence of AGE-mediated disruption of glucose transporter availability at the cellular level. Nevertheless, the absence of insulin stimulation experiments limits the ability to establish dynamic GLUT-4 translocation impairment; these findings should therefore be interpreted as suggestive rather than definitive.

In conclusion, the demonstration that AGEs can directly upregulate IRS-1 in human GCs supports the hypothesis that metabolic byproducts of hyperglycemia and oxidative stress may disrupt ovarian insulin signaling. This finding may be particularly relevant in PCOS, where intrinsic insulin signaling defects coexist with elevated AGE levels. This work also raises the possibility that dietary or pharmacologic interventions targeting AGE formation or RAGE activation could preserve GC insulin responsiveness and support reproductive function. Future studies should investigate the phosphorylation state and activation kinetics of IRS-1 in response to AGEs, assess downstream glucose transport and steroidogenesis, and compare effects between mural and cumulus GCs. These results suggest that AGE exposure may initiate early molecular changes consistent with altered insulin signaling, but do not establish insulin resistance per se. The link to PCOS or insulin resistance should therefore be interpreted as a mechanistic hypothesis. In vivo studies examining the impact of AGE-lowering interventions on ovarian insulin sensitivity and fertility outcomes in women at risk for metabolic disorders could further validate these findings.

## Figures and Tables

**Table 1:** Demographics and clinical characteristics of the participants.

Variable	Mean ± SEM
Age (years)	35.7 ± 1.2
BMI (kg/m^2^)	25.6 ± 1.3
Day 3 FSH (mIU/mL)	7.3 ± 0.9
Antral follicle count (AFC)	15.5 ± 1.4
Total gonadotropin dose per cycle (IU)	3258 ± 364
Duration of stimulation (days)	11.4 ± 0.6
Total oocytes retrieved	12.8 ± 1.4
Mature (MII) oocytes	9.2 ± 1.2
Peak estradiol on day of trigger (pg/mL)	1945.0 ± 210.4

Data expressed as mean ± SEM. BMI, body mass index; FSH, follicle-stimulating hormone; IU, international unit; AFC, antral follicle count; MII, metaphase II. All participants had normal ovarian reserve and no diagnosis of diabetes, endometriosis, or metabolic disorder.

## Data Availability

The data presented in this study are available on request from the corresponding author and are not publicly available due to privacy and ethical restrictions.

## References

[1] Goldberg T., Cai W., Peppa M., Dardaine V., Baliga B.S., Uribarri J., Vlassara H. (2004). Advanced glycoxidation end products in commonly consumed foods. J. Am. Diet. Assoc..

[2] Tantalaki E., Piperi C., Livadas S., Kollias A., Adamopoulos C., Koulouri A., Christakou C., Diamanti-Kandarakis E. (2014). Impact of dietary modification of advanced glycation end products (AGEs) on the hormonal and metabolic profile of women with polycystic ovary syndrome (PCOS). Hormones.

[3] Merhi Z. (2014). Advanced glycation end products and their relevance in female reproduction. Hum. Reprod..

[4] Diamanti-Kandarakis E., Piperi C., Patsouris E., Korkolopoulou P., Panidis D., Pawelczyk L., Papavassiliou A.G., Duleba A.J. (2007). Immunohistochemical localization of advanced glycation end-products (AGEs) and their receptor (RAGE) in polycystic and normal ovaries. Histochem. Cell Biol..

[5] Basta G. (2008). Receptor for advanced glycation endproducts and atherosclerosis: From basic mechanisms to clinical implications. Atherosclerosis.

[6] Monnier V.M., Sell D.R. (2006). Prevention and repair of protein damage by the Maillard reaction in vivo. Rejuvenation Res..

[7] Lander H.M., Tauras J.M., Ogiste J.S., Hori O., Moss R.A., Schmidt A.M. (1997). Activation of the receptor for advanced glycation end products triggers a p21(ras)-dependent mitogen-activated protein kinase pathway regulated by oxidant stress. J. Biol. Chem..

[8] Inagi R. (2011). Inhibitors of advanced glycation and endoplasmic reticulum stress. Methods Enzymol..

[9] Piperi C., Adamopoulos C., Dalagiorgou G., Diamanti-Kandarakis E., Papavassiliou A.G. (2012). Crosstalk between advanced glycation and endoplasmic reticulum stress: Emerging therapeutic targeting for metabolic diseases. J. Clin. Endocrinol. Metab..

[10] Marsche G., Weigle B., Sattler W., Malle E. (2007). Soluble RAGE blocks scavenger receptor CD36-mediated uptake of hypochlorite-modified low-density lipoprotein. FASEB J..

[11] Fujii E.Y., Nakayama M. (2010). The measurements of RAGE, VEGF, and AGEs in the plasma and follicular fluid of reproductive women: The influence of aging. Fertil. Steril..

[12] Malickova K., Jarosova R., Rezabek K., Fait T., Masata J., Janatkova I., Zima T., Kalousova M. (2010). Concentrations of sRAGE in serum and follicular fluid in assisted reproductive cycles—A preliminary study. Clin. Lab..

[13] Bonetti T.C., Borges E., Braga D.P., Iaconelli A., Kleine J.P., Silva I.D. (2013). Intrafollicular soluble receptor for advanced glycation end products (sRAGE) and embryo quality in assisted reproduction. Reprod. Biomed. Online.

[14] Merhi Z., Irani M., Doswell A.D., Ambroggio J. (2014). Follicular fluid soluble receptor for advanced glycation end-products (sRAGE): A potential indicator of ovarian reserve. J. Clin. Endocrinol. Metab..

[15] Basta G., Sironi A.M., Lazzerini G., Del Turco S., Buzzigoli E., Casolaro A., Natali A., Ferrannini E., Gastaldelli A. (2006). Circulating soluble receptor for advanced glycation end products is inversely associated with glycemic control and S100A12 protein. J. Clin. Endocrinol. Metab..

[16] Choi K.M., Han K.A., Ahn H.J., Hwang S.Y., Hong H.C., Choi H.Y., Yang S.J., Yoo H.J., Baik S.H., Choi D.S. (2012). Effects of exercise on sRAGE levels and cardiometabolic risk factors in patients with type 2 diabetes: A randomized controlled trial. J. Clin. Endocrinol. Metab..

[17] Souza A.W., de Leeuw K., van Timmeren M.M., Limburg P.C., Stegeman C.A., Bijl M., Westra J., Kallenberg C.G. (2014). Impact of serum high mobility group box 1 and soluble receptor for advanced glycation end-products on subclinical atherosclerosis in patients with granulomatosis with polyangiitis. PLoS ONE.

[18] Jensen L.J., Flyvbjerg A., Bjerre M. (2015). Soluble Receptor for Advanced Glycation End Product: A Biomarker for Acute Coronary Syndrome. Biomed. Res. Int..

[19] Yonchuk J.G., Silverman E.K., Bowler R.P., Agusti A., Lomas D.A., Miller B.E., Tal-Singer R., Mayer R.J. (2015). Circulating soluble receptor for advanced glycation end products (sRAGE) as a biomarker of emphysema and the RAGE axis in the lung. Am. J. Respir. Crit. Care Med..

[20] Pertynska-Marczewska M., Diamanti-Kandarakis E., Zhang J., Merhi Z. (2015). Advanced glycation end products: A link between metabolic and endothelial dysfunction in polycystic ovary syndrome?. Metabolism.

[21] Pertynska-Marczewska M., Merhi Z. (2015). Relationship of Advanced Glycation End Products With Cardiovascular Disease in Menopausal Women. Reprod. Sci..

[22] Cerami C., Founds H., Nicholl I., Mitsuhashi T., Giordano D., Vanpatten S., Lee A., Al-Abed Y., Vlassara H., Bucala R. (1997). Tobacco smoke is a source of toxic reactive glycation products. Proc. Natl. Acad. Sci. USA.

[23] Diamanti-Kandarakis E., Piperi C., Korkolopoulou P., Kandaraki E., Levidou G., Papalois A., Patsouris E., Papavassiliou A.G. (2007). Accumulation of dietary glycotoxins in the reproductive system of normal female rats. J. Mol. Med..

[24] Kandaraki E., Chatzigeorgiou A., Piperi C., Palioura E., Palimeri S., Korkolopoulou P., Koutsilieris M., Papavassiliou A.G. (2012). Reduced ovarian glyoxalase-I activity by dietary glycotoxins and androgen excess: A causative link to polycystic ovarian syndrome. Mol. Med..

[25] Rutkowska A.Z., Diamanti-Kandarakis E. (2016). Do Advanced Glycation End Products (AGEs) Contribute to the Comorbidities of Polycystic Ovary Syndrome (PCOS)?. Curr. Pharm. Des..

[26] Diamanti-Kandarakis E., Chatzigeorgiou A., Papageorgiou E., Koundouras D., Koutsilieris M. (2016). Advanced glycation end-products and insulin signaling in granulosa cells. Exp. Biol. Med..

[27] Shi X., Xie X., Jia Y., Li S. (2016). Associations of insulin receptor and insulin receptor substrates genetic polymorphisms with polycystic ovary syndrome: A systematic review and meta-analysis. J. Obstet. Gynaecol. Res..

[28] Wu X.K., Zhou S.Y., Liu J.X., Pöllänen P., Sallinen K., Mäkinen M., Erkkola R. (2003). Selective ovary resistance to insulin signaling in women with polycystic ovary syndrome. Fertil. Steril..

[29] Vanderhyden B.C., Tonary A.M. (1995). Differential regulation of progesterone and estradiol production by mouse cumulus and mural granulosa cells by A factor(s) secreted by the oocyte. Biol. Reprod..

[30] Eppig J.J., Chesnel F., Hirao Y., O’Brien M.J., Pendola F.L., Watanabe S., Wigglesworth K. (1997). Oocyte control of granulosa cell development: How and why. Hum. Reprod..

[31] Uyar A., Torrealday S., Seli E. (2013). Cumulus and granulosa cell markers of oocyte and embryo quality. Fertil. Steril..

[32] Dumesic D.A., Meldrum D.R., Katz-Jaffe M.G., Krisher R.L., Schoolcraft W.B. (2015). Oocyte environment: Follicular fluid and cumulus cells are critical for oocyte health. Fertil. Steril..

[33] Merhi Z. (2019). Vitamin D attenuates the effect of advanced glycation end products on anti-Mullerian hormone signaling. Mol. Cell Endocrinol..

[34] Rice S., Pellatt L.J., Bryan S.J., Whitehead S.A., Mason H.D. (2011). Action of metformin on the insulin-signaling pathway and on glucose transport in human granulosa cells. J. Clin. Endocrinol. Metab..

[35] Diamanti-Kandarakis E., Katsikis I., Piperi C., Kandaraki E., Piouka A., Papavassiliou A.G., Panidis D. (2008). Increased serum advanced glycation end-products is a distinct finding in lean women with polycystic ovary syndrome (PCOS). Clin. Endocrinol..

[36] Zhao H., Zhang J., Cheng X., Nie X., He B. (2023). Insulin resistance in polycystic ovary syndrome across various tissues: An updated review of pathogenesis, evaluation, and treatment. J. Ovarian Res..

[37] Aguirre V., Werner E.D., Giraud J., Lee Y.H., Shoelson S.E., White M.F. (2002). Phosphorylation of Ser307 in insulin receptor substrate-1 blocks interactions with the insulin receptor and inhibits insulin action. J. Biol. Chem..

